# An achromatic X-ray lens

**DOI:** 10.1038/s41467-022-28902-8

**Published:** 2022-03-14

**Authors:** Adam Kubec, Marie-Christine Zdora, Umut T. Sanli, Ana Diaz, Joan Vila-Comamala, Christian David

**Affiliations:** 1 Paul Scherrer Institut, Forschungsstrasse 111, 5232 Villigen PSI, Switzerland; 2 Present Address: XRnanotech GmbH, Forschungsstrasse 111, 5232 Villigen PSI, Switzerland

**Keywords:** Imaging techniques, Imaging and sensing, X-rays, Lithography

## Abstract

Diffractive and refractive optical elements have become an integral part of most high-resolution X-ray microscopes. However, they suffer from inherent chromatic aberration. This has to date restricted their use to narrow-bandwidth radiation, essentially limiting such high-resolution X-ray microscopes to high-brightness synchrotron sources. Similar to visible light optics, one way to tackle chromatic aberration is by combining a focusing and a defocusing optic with different dispersive powers. Here, we present the first successful experimental realisation of an X-ray achromat, consisting of a focusing diffractive Fresnel zone plate (FZP) and a defocusing refractive lens (RL). Using scanning transmission X-ray microscopy (STXM) and ptychography, we demonstrate sub-micrometre achromatic focusing over a wide energy range without any focal adjustment. This type of X-ray achromat will overcome previous limitations set by the chromatic aberration of diffractive and refractive optics and paves the way for new applications in spectroscopy and microscopy at broadband X-ray tube sources.

## Abstract (web summary)

X-ray diffractive and refractive optical elements suffer from chromatic aberrations, limiting high-resolution X-ray microscopes mainly to bright synchrotron sources. Here, the authors experimentally realise an achromatic X-ray lens by combing a focusing diffractive Fresnel zone plate and a defocusing refractive lens.

## Introduction

X-ray techniques for the non-invasive investigation of the inner structure and elemental composition of matter at the micro- and nanoscale require high-performance X-ray optics. For this purpose, various types of reflective, refractive, and diffractive optical elements have been developed in the last decades^1–3^. Reflective X-ray optics rely on grazing incidence configuration and require complicated geometries to produce a magnified image of an extended field of view^4,5^, resulting in long focal lengths that are incompatible with a compact setup. While these limitations do not apply to refractive and diffractive optics, making them more suitable for the formation of magnified X-ray images, they suffer from inherent chromatic aberration, meaning that X-rays of different energies are not focused to the same focal plane. As a consequence, the performance of these optics for multi-energy or spectroscopic measurements at synchrotrons and for high-resolution microscopy at polychromatic X-ray tube sources has to date been severely limited.

In the visible-light regime, chromatic aberration of refractive lenses was observed already centuries ago, impairing the performance of telescopes^6^. In the mid-18th century, Chester Moor Hall found a solution for tackling the chromaticity by stacking a focusing lens made of crown glass and a defocusing lens made of flint glass to form an achromatic doublet^7^. Owing to the dispersion being stronger for flint glass than for crown glass, a proper combination of the two lenses provided identical focal lengths for two distinct wavelengths and low chromatic aberration for the wavelength range between them, despite the strong chromaticity of each individual lens. The span between these two wavelengths over which the remaining change in focal length lies within the depth of focus (DOF) is commonly defined as the achromatic range.

The concept of an achromatic doublet can be transferred to the X-ray regime. For X-rays, the refraction and absorption in matter are described by the complex refractive index *n*, which is related to the atomic scattering factor *f*_a_ = *f*_1_ + *if*_2_ of the atoms in the given material:

1 $$n=1-\delta +i\beta =1-\frac{{r}_{e}{n}_{a}{\lambda }^{2}}{2\pi }({f}_{1}+i{f}_{2}),$$

where (1 − *δ*) and *β* are the real and imaginary parts of *n*, *λ* is the X-ray wavelength, *r*_*e*_ the classical radius of the electron, and *n*_*a*_ the number of atoms per volume. For the majority of the X-ray regime, the real part *f*_1_ of the atomic scattering factor changes only little with *λ*, meaning that the material dispersion *D* of *f*_1_ is close to zero, *D* = (Δ*f*_1_/*f*_1_)/(Δ*λ*/*λ*) ≈ 0, and *δ* can be approximated as *δ* ∝ *λ*^2^ for all materials (see Eq. (1)). Only near the absorption edges, *D* reaches large positive or even negative values, leading to anomalous dispersion. Combining two refractive X-ray lenses from different materials therefore cannot provide achromatic behaviour over an extended range of X-ray wavelengths.

**Fig. 1:**
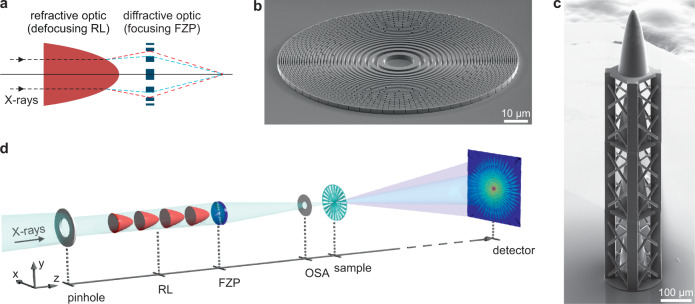
Concept of the X-ray achromat and experimental setup. **a** Principle of achromatic focusing: The chromaticity of the defocusing refractive lens (RL) acts as a corrector for the chromatic behaviour of the focusing Fresnel zone plate (FZP). **b** Scanning electron microscopy (SEM) image of a nickel FZP fabricated by electron-beam lithography and nickel electroplating, as used for the comparison measurements. **c** SEM image of the RL consisting of four stacked paraboloids 3D-printed using two-photon polymerisation lithography. **d** Sketch of the experimental setup for scanning transmission X-ray microscopy (STXM) and ptychography using the achromat as a focusing optic.

Refractive and diffractive X-ray lenses, on the other hand, scale differently regarding their chromaticity, which opens up the fundamental possibility of combining them to compensate for chromatic aberration and form an X-ray achromat^8,9^, see Fig. 1a. In the theoretical work by Wang et al.^8^, a general expression providing achromatic behaviour is derived for the ratio of the focal lengths *f*_RL_ of the RL and *f*_FZP_ of the diffractive FZP:

2 $$\frac{{f}_{{{{{{{{\rm{RL}}}}}}}}}}{{f}_{{{{{{{{\rm{FZP}}}}}}}}}}=-(2+D)$$

A dispersion of *D* ≪ − 2 can be found for wavelengths near the absorption edges in the extreme ultra-violet and the soft X-ray regimes. Wang et al. conclude that one can thus form an achromat by combining a focusing FZP with a weakly focusing RL. This elegant solution, however, is limited to narrow wavelength ranges where a material suitable for the fabrication of the refractive corrector shows strongly negative dispersion. In the broad wavelength ranges far from the absorption edges, where *D* ≈ 0, the focal length of a FZP is proportional to the inverse wavelength, *f*_FZP_ ∝ *λ*^−1^, whereas the focal length of a RL scales as *f*_RL_ ∝ *λ*^−2^. Therefore, according to Eq. (2), an X-ray achromat needs to be composed of a focusing diffractive part (FZP), see Fig. 1b, and a defocusing refractive part (RL), see Fig. 1c, fulfilling the relation *f*_RL_ = −2*f*_FZP_. The focal length *f*_A_ of such an achromat is then given by: *f*_A_ = 2*f*_FZP_. These relations are valid for the case of direct contact of the two elements. When taking into account their separation along the beam direction, we arrive at slightly modified expressions, see Methods and Supplementary Material. This type of X-ray achromat has previously been proposed in the context of telescopes in X-ray astronomy^10–12^. More recently, the promising potential of X-ray achromats has been discussed for microscopy and spectroscopy and moreover for focusing of short pulses without distortion in time^9,13,14^. However, an experimental realisation has not been reported to date.

Here, we present a compact optical system that can achieve achromatic focusing, delivering images of consistently high quality, over an X-ray photon energy range from 5.8 keV to 7.3 keV.

## Results

The presented X-ray achromat consisted of a FZP fabricated using electron-beam lithography and nickel electroplating and a 3D-printed RL made by two-photon polymerisation. The achromat was installed as a focusing optic at the cSAXS beamline of the Swiss Light Source (Villigen, Switzerland) with the experimental setup shown in Fig. 1d. The fabrication, properties, and arrangement of the optical components are described in more detail in the Methods section. In order to demonstrate the achromatic behaviour of the optic, STXM as well as ptychography measurements were performed at multiple X-ray energies between 5.2 keV and 8.0 keV, with the sample placed in the focal plane of the achromat at its optimum energy of 6.4 keV. For comparison, reference measurements were taken with a conventional FZP (see Fig. 1b) instead of the achromat. Both optics had the same numerical aperture, limiting the achievable spot size to about 500 nm.

**Fig. 2:**
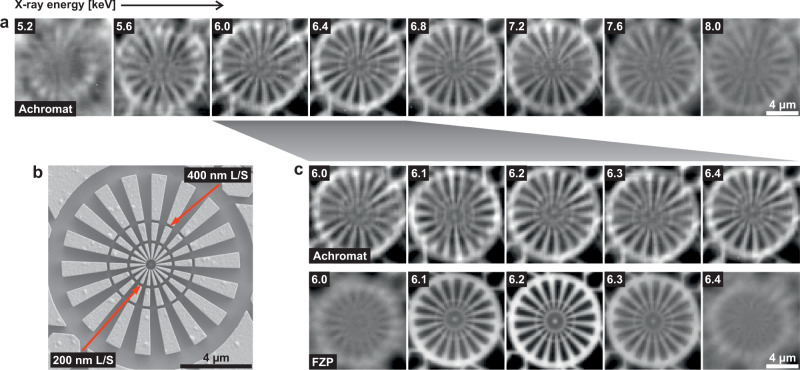
Demonstration of STXM imaging at different energies using the achromat. **a** STXM images of the Siemens star sample shown in panel b obtained with the achromat, indicating an achromatic range of  > 1 keV around the optimum energy of ~ 6.4 keV. **b** SEM image of the Siemens star test sample. The radial lines and spaces (L/S) at the outer and inner concentric rings have widths of 400 nm and 200 nm, respectively, see red arrows. **c** Comparison of the STXM results in the energy range of 6.0 keV to 6.4 keV obtained with the achromat (top) and the conventional FZP (bottom). While the contrast of the FZP images changes rapidly with the energy, the image quality achieved with the achromat varies only little.

Figure 2a presents the STXM images of the Siemens star test sample shown in Fig. 2b acquired with the achromat as a focusing optic at different X-ray energies. No significant change of the image quality is visible between 6.0 keV and 7.2 keV. The significant advantage of using the achromat is clear when directly comparing its performance with STXM data obtained with the FZP as an optical element, see Fig. 2c. While the achromat delivers images of consistently high contrast and spatial resolution over a wide energy range, the images taken with the FZP exhibit severe blurring already at X-ray energies deviating 200 eV from its design energy of 6.2 keV. The achromat is capable of resolving line widths below 400 nm over its achromatic range, however, it does not reach the image quality of the FZP at its design energy. This is due to shape imperfections in the refractive element resulting in aberrations and will be improved in future designs of the achromat.

**Fig. 3:**
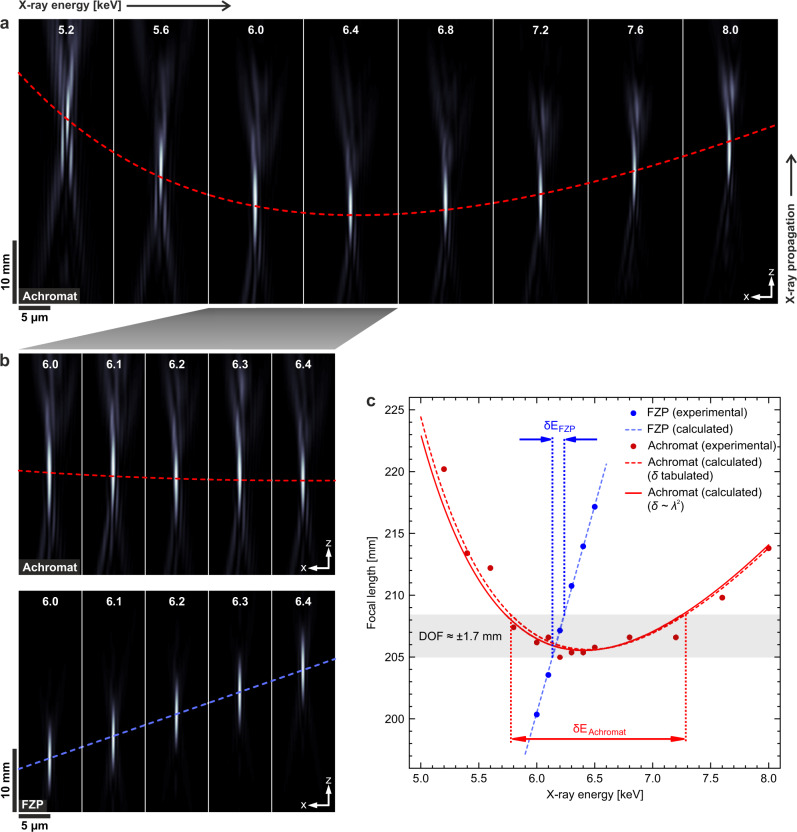
Evolution of the X-ray beam profile with the energy measured with ptychography. **a** Caustics at energies from 5.2 keV to 8.0 keV obtained with the achromat. The red dashed line indicates the location of the focal plane at the different energies. **b** Comparison of the caustics obtained with the achromat and the FZP. While the position of the focal plane remains almost constant with the energy for the achromat (red dashed line), it changes rapidly for the FZP (blue dashed line). **c** Calculated curves (solid and dashed lines) and experimental data (dots) for the focal length versus energy for the FZP (blue) and the achromat (red; solid: based on Eq. (6), dashed: based on tabulated refractive index values for the calculation of *f*_RL_ at each energy).

The focusing properties of the achromat can also be analysed by looking at the evolution of the X-ray wavefield with the energy. For this purpose, ptychography measurements (see Supplementary Fig. 1 in the Supplementary Material) were conducted at the same energies as the STXM data, which allows for retrieving the illuminating X-ray wavefield. The intensity distribution of the wavefield at the sample position was then propagated computationally along the optical axis *z* to create an X-ray beam caustic at each energy. Cuts along the *xz* plane, see coordinate system in Fig. 1d, through the caustics of the achromat are shown in Fig. 3a, where the focus position is indicated with a red dashed line. A direct comparison of the caustics obtained with the achromat and the FZP, see Fig. 3b, confirms the significant gain in achromatic range achieved by the achromat, as already observed in the STXM images in Fig. 2.

The experimental results for the location of the focal spot agree well with the corresponding calculated curves for both the achromat and the FZP, see Fig. 3c. The difference between the solid and dashed red curves for the achromat, which were calculated using Eq. (6) and inserting tabulated *δ* and *β* values^15^ into Eq. (4), respectively, is small. This indicates that the assumption of no material dispersion that was made in Eq. (6) holds well, see Methods. Looking at Fig. 3c, we expect in-focus imaging within the wide achromatic range *δE*_Achromat_ from about 5.8 keV to 7.3 keV for the achromat but only within the relatively narrow range *δE*_FZP_ ≈ 100 eV for the FZP, which is consistent with the results shown in Fig. 2.

**Fig. 4:**
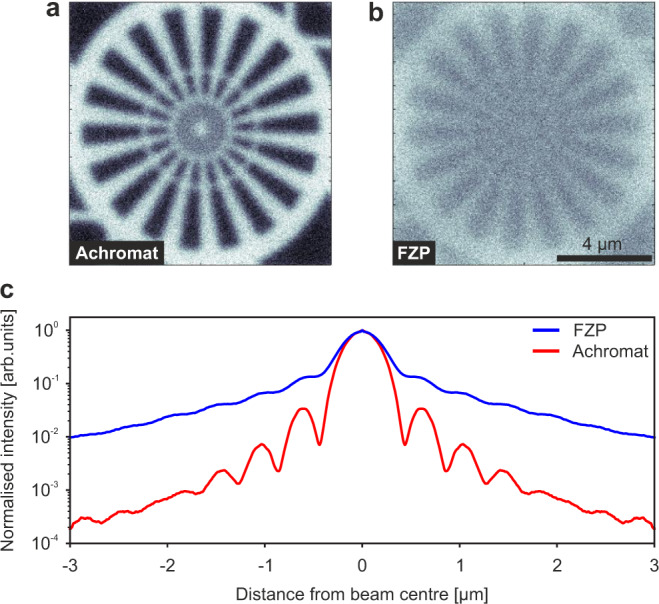
Simulations of polychromatic X-ray focusing with an achromatic lens and with a single FZP (energy range from 5.6 keV to 6.8 keV). **a** Convolution of the simulated polychromatic X-ray beam of the achromat with the binarised image of the Siemens star in Fig. 2b. **b** Convolution of the same image with the simulated polychromatic X-ray beam of the FZP. Gaussian noise was added to both images after convolution to model the image noise in experimental data. **c** Line profiles of the simulated polychromatic X-ray beams (normalised to central peak) for the achromat (red) and the FZP (blue).

To demonstrate the potential of the presented achromat for imaging with polychromatic X-rays, we performed wavefield propagation simulations with an achromat and a single FZP, see Methods. Comparing the simulated data allows for assessing the gain in image quality over a FZP that is attainable with an optimised achromat without the effects manufacturing errors. Figure 4a shows the result of a convolution of the binarised SEM image of the Siemens star in Fig. 2b with the simulated polychromatic X-ray beam with an energy range between 5.6 keV and 6.8 keV obtained with an achromat. The gain in image quality compared to the image in Fig. 4b simulated analogously using a FZP as an optical element is striking. The image contrast is significantly improved with the achromat, which in the presence of noise enables the visualisation of much smaller features. The improvement in image quality can be understood from the simulated polychromatic beam profiles in Fig. 4c, where much stronger side lobes of the beam can be observed for the FZP than for the achromat.

## Discussion

We have presented the experimental realisation of an X-ray achromat consisting of a focusing diffractive and a defocusing refractive optical element, which achieves steady imaging performance over a wide energy range without change of the focal settings. This feature will facilitate experiments with monochromatic X-rays that rely on rapid or frequent change of photon energy. Moreover, it will allow for more efficient use of broad-band radiation in X-ray microscopy and nanoanalysis, including the diffraction-limited focusing of the full radiation from an undulator at synchrotron or X-ray laser facilities. The presented achromat will be of particular benefit for use at polychromatic laboratory X-ray tube sources, as indicated by our simulations, which will enable efficient high-resolution microscopy at laboratory-based X-ray systems. Using chromatic lenses as available to date, the available spectrum had to be cut down to a narrow X-ray bandwidth in previous setups, which severely reduced the photon flux and hence led to long scan times and poor statistics. For the same reason, we expect the proposed achromatic optic to unfold microscopy applications for neutron beams^16^, as theoretically explored in a previous publication^17^.

The presented work describes the proof-of-principle of an X-ray achromat, leaving room for further improvements through advances in manufacturing techniques and design. Reducing the X-ray spot size will require RLs with stronger refracting power and thus higher structures with smaller radii of curvature. This challenge, along with the significant absorption losses in the RL, can be addressed by designing the RL with a stepped profile, as suggested previously^8^. Furthermore, while in its current implementation the diffractive and the refractive parts of the achromat are mounted on two separate support membranes, we will explore the fabrication as a single monolithic device in the future. This will simplify the alignment and use of the achromatic X-ray lens at the experimental setup.

## Methods

### Fabrication of the Fresnel zone plates

The FZP that was used as part of the achromat was a double-sided blazed FZP with four steps. It consisted of nickel structures on the top and bottom of a 250 nm-thick silicon nitride (Si_3_N_4_) membrane, which were aligned with respect to each other and had heights of 1 μm (back) and 2.2 μm (front). The FZP had a diameter of 100 μm and an effective outermost zone width of 200 nm. The diffraction efficiency of the FZP was measured to be 54.7% at its design energy of 6.2 keV. The FZP used for the comparison measurements was binary and had a diameter of 100 μm and an outermost zone width of 415 nm. It consisted of 2.2 μm-high nickel structures on a 250 nm-thick Si_3_N_4_ membrane with a theoretical efficiency of 36.8% at 6.2 keV^15^. For the fabrication of both FZPs, the membranes were first covered with a chromium-gold-chromium plating base by evaporation and then spin-coated with a Poly(methyl methacrylate) (PMMA) resist layer, which was subsequently exposed by electron-beam lithography (Vistec EBPG 5000+ electron-beam writer at 100 kV acceleration voltage). The patterns were developed, resist trenches cleaned by plasma etching, and then filled with nickel via electroplating, which was followed by removal of the residual PMMA. For the blazed FZP, this process was performed for both sides of the chip. More details on the fabrication process of the FZPs can be found in earlier publications^18,19^.

### Fabrication of the refractive lens

The RL was 3D-printed with two-photon polymerisation-induced lithography using a Nanoscribe Photonic Professional GT^20^. The flexible capabilities of two-photon polymerisation have been exploited previously for the fabrication of various types of X-ray optics such as compound RLs for focusing and phase plates for wavefront correction^14,21^.

The manufacturing process of the RL was similar to the methods described by Lyubomirskiy et al.^21^. Nanoscribe IP-S resist in dip-in lithography mode with a 25× objective was used. The total writing time was approximately 17 h. The RL was printed in vertical geometry on a 250 nm-thick Si_3_N_4_ membrane and consisted of a stack of *N* = 4 individual paraboloid elements of *R* = 5.3 μm apex radius and 236 μm height each. Its focal length *f*_RL_ is hence given by:

3 $${f}_{{{{{{{{\rm{RL}}}}}}}}}=\frac{R}{N\delta }$$

Figure 1c shows an SEM image of the RL used in the experiment. Vertical support structures were printed to stack the four paraboloids. The X-ray absorption and diffraction properties of the resist can be estimated using the material compound C_14_H_18_O_7_ with a density of 1.2 g/cm^3^^15^. The transmission through the RL calculated using these properties is 23% at 5.2 keV, 44% at 6.2 keV, and 73% at 8.0 keV.

### Properties of the achromat

The achromat was designed in a way that the RL acted as a corrector for the chromaticity of the FZP. The efficiency of such an achromat can be approximated by the product of the diffraction efficiency of the FZP and the X-ray transmission through the RL, which results in a theoretical efficiency of about 24% at the design energy of 6.2 keV for our optics.

The FZP and the RL were separated by a distance *d* = 1 mm along the optical axis (RL upstream of FZP), making the approximation in Eq. (2), which holds for *d* = 0, not strictly valid. In the case of *d* ≠ 0, the focal length of the achromat can be defined using the concept of the back focal length (BFL) *l*_*i*_ of a lens combination^22^:

4 $${l}_{i}=\frac{{f}_{{{{{{{{\rm{FZP}}}}}}}}}(d-{f}_{{{{{{{{\rm{RL}}}}}}}}})}{d-({f}_{{{{{{{{\rm{FZP}}}}}}}}}+{f}_{{{{{{{{\rm{RL}}}}}}}}})}$$

The BFL is the position of the focus measured from the downstream optical element, here the FZP. The energy dependence of *l*_*i*_(*E*) can be described by considering the energy dependences of *f*_FZP_(*E*) and *f*_RL_(*E*):

5 $${f}_{{{{{{{{\rm{FZP}}}}}}}}}(E) 	={f}_{D}\left(1+\left[\frac{{{\Delta }}E}{{E}_{0}}\right]\right)\\ {f}_{{{{{{{{\rm{RL}}}}}}}}}(E) 	={f}_{R}{\left(1+\left[\frac{{{\Delta }}E}{{E}_{0}}\right]\right)}^{2},$$

where *E*_0_ is the reference energy (optimum energy of the achromat), Δ*E* is the energy difference to *E*_0_ and *f*_*D*_ = *f*_FZP_(*E*_0_), and *f*_*R*_ = *f*_RL_(*E*_0_) the focal lengths at *E*_0_. This leads to the expression:

6 $${l}_{i}(E)=	\, \frac{{f}_{{{{{{{{\rm{D}}}}}}}}}(2{f}_{{{{{{{{\rm{D}}}}}}}}}+d)}{{f}_{{{{{{{{\rm{D}}}}}}}}}+d}\left[1+\frac{\left(4d{f}_{{{{{{{{\rm{D}}}}}}}}}+{d}^{2}\right)}{(2{f}_{{{{{{{{\rm{D}}}}}}}}}+d)({f}_{{{{{{{{\rm{D}}}}}}}}}+d)}\left[\frac{{{\Delta }}E}{{E}_{0}}\right]\right.\\ 	+\frac{\left(2{{f}_{{{{{{{{\rm{D}}}}}}}}}}^{3}-6d{{f}_{{{{{{{{\rm{D}}}}}}}}}}^{2}+{d}^{2}{f}_{{{{{{{{\rm{D}}}}}}}}}\right)}{(2{f}_{{{{{{{{\rm{D}}}}}}}}}^{2}+3d{f}_{{{{{{{{\rm{D}}}}}}}}}+{d}^{2})({f}_{{{{{{{{\rm{D}}}}}}}}}+d)}{\left[\frac{{{\Delta }}E}{{E}_{0}}\right]}^{2}\\ 	\left.-\frac{\left(4{{f}_{{{{{{{{\rm{D}}}}}}}}}}^{4}-16d{{f}_{{{{{{{{\rm{D}}}}}}}}}}^{3}+7{d}^{2}{{f}_{{{{{{{{\rm{D}}}}}}}}}}^{2}\right)}{(2{{f}_{{{{{{{{\rm{D}}}}}}}}}}^{3}+5d{{f}_{{{{{{{{\rm{D}}}}}}}}}}^{2}+4{d}^{2}{f}_{{{{{{{{\rm{D}}}}}}}}}+{d}^{3})({f}_{{{{{{{{\rm{D}}}}}}}}}+d)}{\left[\frac{{{\Delta }}E}{{E}_{0}}\right]}^{3}+...\right],$$

which is derived in the Supplementary Material.

The solid red curve in Fig. 3c represents the BFL values calculated according to Eq. (6). The dashed red curve in Fig. 3c was obtained using tabulated values of the refractive index of the RL material at the different energies^15^. The slight difference between the two curves is due to the fact that the tabulated values take into account the material dispersion, whereas in Eq. (6) it is assumed to be negligible (*D* ≈ 0). For the X-ray energy range in our experiment, far from the absorption edges, this approximation holds well and the difference between the two curves is small.

### Fabrication of the Siemens star test object

The test pattern was a Siemens star made from 750 nm-high gold structures. It had a diameter of 10 μm and consisted of 18 spokes with inner spoke width of 50 nm and outer spoke width of 873 nm. The Siemens star was fabricated on a 250 nm-thick Si_3_N_4_ membrane with similar process steps as the FZPs, using electron-beam lithography and electroplating.

### Experimental setup

Experiments were performed at the cSAXS beamline of the Swiss Light Source at the Paul Scherrer Institute, Villigen, Switzerland. Monochromatic X-rays of different energies were extracted from the undulator spectrum with a silicon double-crystal monochromator (relative energy bandwidth:~10^−4^). The experimental setup for the measurements is sketched in Fig. 1d. The beam-shaping slits upstream of the setup were set to a position that ensured fully coherent illumination of the lens aperture. Further downstream, the incident X-ray beam was reduced to the size of the lens aperture by a pinhole of 100 μm diameter. The beam was then focused with the achromat consisting of the RL and the blazed FZP that were separated by approximately 1 mm along the beam direction. Undesired diffraction orders of the FZP were removed by an order-sorting aperture (OSA) of 30 μm diameter mounted downstream of the achromat. The X-ray probe shaped this way illuminated a Siemens star test object, and the resulting diffraction pattern was recorded by a photon-counting detector located approximately 7 m downstream of the sample.

### STXM measurements

STXM^23,24^ was performed by transversely raster-scanning the sample on a grid of 60 × 60 steps with a step size of 200 nm, recording a diffraction pattern at each position using a Pilatus 2M single-photon counting detector (chip size: 1475 × 1679 pixels, pixel size:172 × 172 μm^2^). The sample was positioned in the focal plane of the achromat at an energy of 6.4 keV. The exposure time for each recorded frame was 200 ms. Each pixel of the reconstructed STXM image was created by integrating the photon counts in the corresponding diffraction pattern over a region of interest of 192 × 192 pixels around the centre of the beam.

### Ptychography measurements

In the last decade, X-ray ptychography^25,26^ has been identified as a powerful tool for characterising X-ray optics^27–29^. The method was used here to retrieve the caustics of the X-ray beam illuminating the sample. The Siemens star test pattern was scanned over an area of 4 × 4 μm^2^ following a Fermat spiral pattern with a step size of 250 nm and the diffraction patterns were recorded with an Eiger 500k single-photon counting detector (chip size: 512 × 1024 pixels, pixel size: 75 × 75 μm^2^). The exposure time for each recorded pattern was 200 nm. The X-ray probe was retrieved from the measurements using the ptychographic reconstruction framework PtychoShelves^30^. The probe intensity was then numerically free-space propagated using an angular-spectrum propagator implemented in PtychoShelves. From the beam caustics retrieved this way, information on the position of the focal plane and the size of the focal spot was extracted at each X-ray energy for the achromat as well as the single FZP. The measured data points for the focal length in Fig. 3c represent the positions of the voxel with highest intensity in each caustic.

### Polychromatic X-ray propagation simulations

Simulations of X-ray focusing were performed for both an achromat, consisting of a RL and a FZP, and a single FZP. In-house developed code in Matlab was used for the simulations, which is based on an angular-spectrum approach described in earlier publications^31^.

The RL was modelled as a single lens with one refraction interface and a diameter of 100 μm, a height of 904 μm and an apex radius of 1.38 μm, made of a material with chemical formula C_14_H_18_O_7_ and density of 1.2 g/cm^3^, see above. It was sliced 100 times using multi-slice propagation^32,33^. The simulated FZP consisted of 2.2 μm-high nickel structures. It had a diameter of 100 μm and an outermost zone width of 415 nm. To obtain focusing in the correct plane, the separation of the two elements in the simulation was set to the physical separation of 1 mm plus the thickness of the RL, giving a total of 1.904 mm.

The simulations were performed for energies in the range of 5.6 keV and 6.8 keV in steps of 50 eV. The X-ray probes obtained this way for both the achromat and the FZP were used to create simulated X-ray images of a Siemens star. For this, the SEM image of the Siemens star in Fig. 2b was binarised and then convolved with the simulated X-ray probe in the focal plane. Subsequently, Gaussian noise with a variance of 0.01 was added to the normalised images to better represent the properties of experimental data.

## Supplementary information

Supplementary Information

## Data Availability

The data generated in this study are available under restricted access due to pending further analysis, access can be obtained from corresponding author M.-C.Z. upon reasonable request.

## Code availability

The code used for the reconstruction of the STXM data, the ptychography data, and for the propagation of the wavefront obtained with ptychography was developed by the cSAXS beamline at the Swiss Light Source and is freely available at https://www.psi.ch/en/sls/csaxs/software.
