# Supplementary material for: An achromatic X-ray lens
Source: Nat Commun. 2022 Mar 14;13:1305. doi: 10.1038/s41467-022-28902-8 (PMC8921332; doi:10.1038/s41467-022-28902-8)
Supplement: Supplementary file 1 — Supplementary Information [file 41467_2022_28902_MOESM1_ESM.pdf]

An achromatic X-ray lens – Supplementary Material

Adam Kubec[1,†], Marie-Christine Zdora[1,*], Umut T. Sanli[1], Ana Diaz[1], Joan Vila-Comamala[1], and Christian David[1]

[1]Paul Scherrer Institut, Forschungsstrasse 111, 5232 Villigen PSI, Switzerland.
[†]Present address: XRnanotech GmbH, Forschungsstrasse 111, 5232 Villigen PSI, Switzerland.
[*]Email: marie.zdora@psi.ch

1 Mathematical derivation of the focal length of the achromat

In the following, an expression for the location of the focal plane of an achromat consisting of a refractive and diffractive optical element is derived, taking into account the energy dependence of their focal lengths. We assume that the refractive lens (RL) is placed upstream of the diffractive Fresnel zone plate (FZP), as it was the case in our experiment. This configuration should generally be preferred as it leads to a higher numerical aperture of the system.[1] In the following, we consider the RL and FZP to be a thin lens. The generalised case of a thick RL is described in detail by Chapman et al.[1]

Far from the absorption edges, the focal length f_{FZP} of a FZP and the focal length f_{RL} of a RL can be approximated as functions of the photon energy E as follows:

$$\begin{aligned} f_{\mathrm{FZP}}(E) &= f_D \left[\frac{E}{E_0}\right] \\ f_{\mathrm{RL}}(E) &= f_R \left[\frac{E}{E_0}\right]^2, \end{aligned} \tag{S.1}$$

where $f_{\mathrm{D}} = f_{\mathrm{FZP}}(E_0)$ and $f_{\mathrm{R}} = f_{\mathrm{RL}}(E_0)$ are the focal lengths of the FZP and the RL, respectively, at a given energy E_0. Introducing $E = E_0 + \Delta E$ gives:

$$\begin{aligned} f_{\mathrm{FZP}}(E) &= f_D \left(1 + \left[\frac{\Delta E}{E_0}\right]\right) \\ f_{\mathrm{RL}}(E) &= f_R \left(1 + 2\left[\frac{\Delta E}{E_0}\right] + \left[\frac{\Delta E}{E_0}\right]^2\right) \end{aligned} \tag{S.2}$$

1.1 Case: d = 0

Let us first consider the special case that the RL and FZP are in close contact (separation $d = 0$). The focal length f_{A} of the achromat can then be obtained from the focal lengths of the RL and FZP with the simple relation:

$$\frac{1}{f_{\mathrm{A}}(E)} = \frac{1}{f_{\mathrm{RL}}(E)} + \frac{1}{f_{\mathrm{FZP}}(E)} = \frac{f_{\mathrm{RL}}(E) + f_{\mathrm{FZP}}(E)}{f_{\mathrm{FZP}}(E) f_{\mathrm{RL}}(E)} \tag{S.3}$$

Introducing the energy dependence by substituting Eq. S.2 in Eq. S.3, we obtain:

$$\frac{1}{f_{\mathrm{A}}(E)} = \frac{1}{f_R\left(1+2\left[\frac{\Delta E}{E_0}\right]+\left[\frac{\Delta E}{E_0}\right]^2\right)} + \frac{1}{f_D\left(1+\left[\frac{\Delta E}{E_0}\right]\right)} \tag{S.4}$$

We can now expand Eq. S.4 about $\left[\frac{\Delta E}{E_0}\right]$ by making use of the Taylor series $\frac{1}{1+x} = \sum_{k=0}^{\infty}(-1)^k x^k$ for $x < |1|$ and $\frac{1}{(1+x)^2} = \sum_{k=0}^{\infty}(k+1)(-1)^k x^k$:

$$\frac{1}{f_{\mathrm{A}}(E)} = \frac{1}{f_{\mathrm{R}} f_{\mathrm{D}}} \sum_{k=0}^{\infty} (-1)^k \Big(f_{\mathrm{R}} + (k+1) f_{\mathrm{D}}\Big) \left[\frac{\Delta E}{E_0}\right]^k \tag{S.5}$$

It can be seen that for $f_{\mathrm{R}} = -2f_{\mathrm{D}}$ the linear dependence on $\left[\frac{\Delta E}{E_0}\right]$ cancels and we achieve achromatic focusing, as described by Wang et al. [2] The expression of Eq. S.5 simplifies to:

$$\frac{1}{f_{\mathrm{A}}(E)} = \frac{1}{2f_{\mathrm{D}}} \sum_{k=0}^{\infty} (-1)^k \Big(1-k\Big) \left[\frac{\Delta E}{E_0}\right]^k \tag{S.6}$$

Inverting the formula and using a Taylor series expansion, one obtains the following expression for the focal length $f_{\mathrm{A}}(E)$ of an achromat consisting of a combination of a RL and a FZP that are in direct contact:

$$\begin{aligned} f_{\mathrm{A}}(E) &= 2f_{\mathrm{D}}\left[1 - \sum_{k=0}^{\infty}(-1)^{k+1}2^k \left[\frac{\Delta E}{E_0}\right]^{k+2}\right] \\ &= 2f_{\mathrm{D}}\left[1 + \left[\frac{\Delta E}{E_0}\right]^2 - 2\left[\frac{\Delta E}{E_0}\right]^3 + 4\left[\frac{\Delta E}{E_0}\right]^4 - 8\left[\frac{\Delta E}{E_0}\right]^5 + 16\left[\frac{\Delta E}{E_0}\right]^6 - 32\left[\frac{\Delta E}{E_0}\right]^7 + ...\right] \end{aligned} \tag{S.7}$$

This distance is equivalent to the location of the source image created by this achromat when the source is at infinity.

1.2 Case: d $\neq$ 0

Let us now consider the case in which the two optical elements are separated by a distance $d \neq 0$, with the source at infinity. The image of the source will be located at a distance $l_i(E)$ from the downstream optical element, which is known as **back focal length (BFL)**. The BFL $l_i(E)$ of the achromat can be obtained through the relation:[3]

$$\frac{1}{l_i(E)} = \frac{1}{f_{\mathrm{RL}}(E)-d} + \frac{1}{f_{\mathrm{FZP}}(E)} = \frac{d - (f_{\mathrm{RL}}(E) + f_{\mathrm{FZP}}(E))}{f_{\mathrm{FZP}}(E)(d - f_{\mathrm{RL}}(E))} \tag{S.8}$$

For the case of $d = 0$ the BFL $l_i(E)$ is equivalent to the focal length $f_{\mathrm{A}}(E)$. We can again introduce the energy dependence in terms of $\left[\frac{\Delta E}{E_0}\right]$:

$$\frac{1}{l_i(E)} = \frac{1}{f_R\left(1+2\left[\frac{\Delta E}{E_0}\right]+\left[\frac{\Delta E}{E_0}\right]^2\right) - d} + \frac{1}{f_D\left(1+\left[\frac{\Delta E}{E_0}\right]\right)} \tag{S.9}$$

A Taylor expansion series delivers:

$$\frac{1}{l_i(E)} = \frac{d-(f_\mathrm{R}+f_\mathrm{D})}{(d-f_\mathrm{R})\,f_\mathrm{D}} - \frac{\left(2f_\mathrm{D}f_\mathrm{R}+d^2-2f_\mathrm{R}d+{f_\mathrm{R}}^2\right)}{\left(d^2-2f_\mathrm{R}d+{f_\mathrm{R}}^2\right)f_\mathrm{D}}\left[\frac{\Delta E}{E_0}\right] - \frac{\left(\left(f_\mathrm{R}d+3{f_\mathrm{R}}^2\right)f_\mathrm{D}-d^3+3f_\mathrm{R}d^2-3{f_\mathrm{R}}^2d+{f_\mathrm{R}}^3\right)}{\left(d^3-3f_\mathrm{R}d^2+3{f_\mathrm{R}}^2d-{f_\mathrm{R}}^3\right)f_\mathrm{D}}\left[\frac{\Delta E}{E_0}\right]^2 - \frac{\left(\left(4{f_\mathrm{R}}^2d+4{f_\mathrm{R}}^3\right)f_\mathrm{D}+d^4-4f_\mathrm{R}d^3+6{f_\mathrm{R}}^2d^2-4{f_\mathrm{R}}^3d+{f_\mathrm{R}}^4\right)}{\left(d^4-4f_\mathrm{R}d^3+6{f_\mathrm{R}}^2d^2-4{f_\mathrm{R}}^3d+{f_\mathrm{R}}^4\right)f_\mathrm{D}}\left[\frac{\Delta E}{E_0}\right]^3 + \ldots \tag{S.10}$$

The term in Eq. S.10 that scales linearly with the energy disappears when:

$$f_\mathrm{R}^2 + 2(f_\mathrm{D}-d)f_\mathrm{R} + d^2 = 0, \tag{S.11}$$

which is fulfilled for (considering only the physically meaningful of the two solutions):

$$f_\mathrm{R} = -f_\mathrm{D} + d - \sqrt{f_\mathrm{D}^2 - 2f_\mathrm{D}d} \tag{S.12}$$

Note that for $d = 0$ this gives $f_\mathrm{R} = -2f_\mathrm{D}$, which is exactly the relation derived in subsection 1.1.

Despite the small separation between the optical elements in our experiment, their focal lengths were chosen as $f_\mathrm{R} = -2f_\mathrm{D}$. In this case, Eq. S.12 simplifies to:

$$\frac{1}{l_i(E)} = \frac{f_\mathrm{D}+d}{f_\mathrm{D}(2f_\mathrm{D}+d)}\left[1 - \frac{\left(d^2+4f_\mathrm{D}d\right)}{(d+2f_\mathrm{D})(f_\mathrm{D}+d)}\left[\frac{\Delta E}{E_0}\right] + \frac{\left(d^3+6f_\mathrm{D}d^2+14{f_\mathrm{D}}^2d-4{f_\mathrm{D}}^3\right)}{(d^2+4f_\mathrm{D}d+4{f_\mathrm{D}}^2)(f_\mathrm{D}+d)}\left[\frac{\Delta E}{E_0}\right]^2 - \frac{\left(d^4+8f_\mathrm{D}d^3+24{f_\mathrm{D}}^2d^2+48{f_\mathrm{D}}^3d-16{f_\mathrm{D}}^4\right)}{(d^3+6f_\mathrm{D}d^2+12{f_\mathrm{D}}^2d+8{f_\mathrm{D}}^3)(f_\mathrm{D}+d)}\left[\frac{\Delta E}{E_0}\right]^3 + \ldots\right] \tag{S.13}$$

Inverting the formula and using another Taylor series expansion, we arrive at the final expression for the BFL $l_i(E)$ in our experiment:

$$l_i(E) = \frac{f_\mathrm{D}(2f_\mathrm{D}+d)}{f_\mathrm{D}+d}\left[1 + \frac{\left(4df_\mathrm{D}+d^2\right)}{(2f_\mathrm{D}+d)(f_\mathrm{D}+d)}\left[\frac{\Delta E}{E_0}\right] + \frac{\left(2{f_\mathrm{D}}^3-6d{f_\mathrm{D}}^2+d^2f_\mathrm{D}\right)}{(2f_\mathrm{D}^2+3df_\mathrm{D}+d^2)(f_\mathrm{D}+d)}\left[\frac{\Delta E}{E_0}\right]^2 - \frac{\left(4{f_\mathrm{D}}^4-16d{f_\mathrm{D}}^3+7d^2{f_\mathrm{D}}^2\right)}{(2{f_\mathrm{D}}^3+5d{f_\mathrm{D}}^2+4d^2f_\mathrm{D}+d^3)(f_\mathrm{D}+d)}\left[\frac{\Delta E}{E_0}\right]^3 + \ldots\right] \tag{S.14}$$

Although the linear term in Eq. S.14 does not fully cancel in our case with $d \neq 0$, the first-order coefficient is small compared to the quadratic and higher-order terms. Hence, the difference in focusing behaviour compared to an achromat with zero linear contribution is little. Generally, achromatic focusing can be observed when the linear term in Eq. S.10 is small compared to the higher-order terms.

Analogously to achromatic focusing, one can create the conditions for apochromatic focusing by choosing the right combination of parameters for the focal lengths of the two optical elements and their separation distance d to cancel both the linear and quadratic terms in Eq. S.10, which leads to an identical focal length of the achromat

for three distinct wavelengths. More details on the conditions for apochromatic focusing can be found in the recent publication by Chapman et al.[1]

2 Ptychography results

As described in the main text, ptychography measurements were conducted to obtain information about the focusing properties of the achromat and the FZP. Ptychography delivers both the probe and the object information simultaneously from the same data set. Figure 1 shows the reconstructions of the Siemens star object and the illuminating X-ray probe for the achromat (Supplementary Fig. 1a-c) at its optimum energy and the FZP (Supplementary Fig. 1d-f) at its design energy, obtained using the PtychoShelves software.[4]

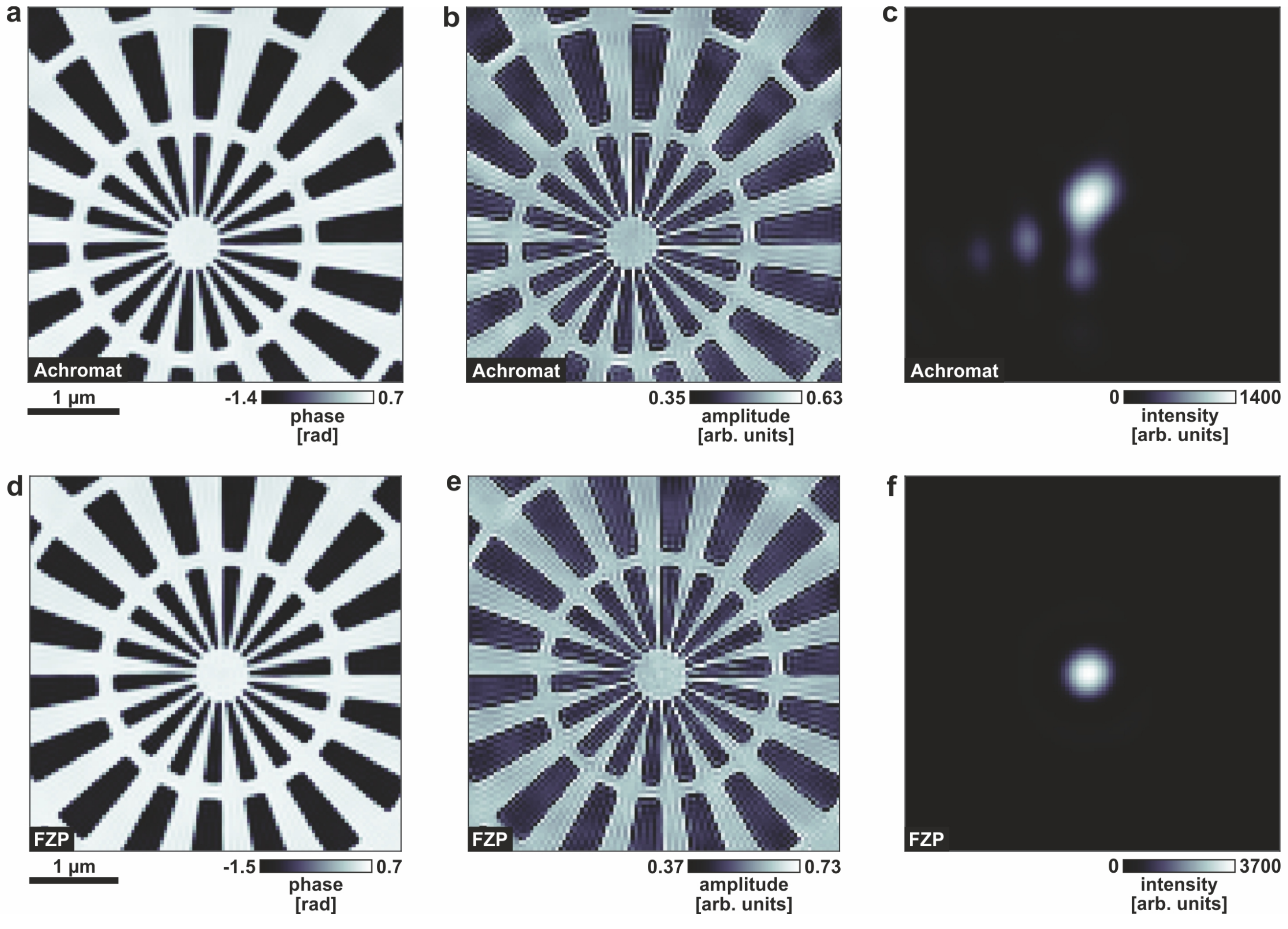

Supplementary Fig. 1 Ptychography results obtained with the achromat and the comparison FZP as focusing optical elements. Reconstruction of **a** the phase, **b** the amplitude of the Siemens star sample in the scanned region and **c** intensity of the illuminating X-ray probe obtained from the ptychographic data acquired with the achromat at an X-ray energy of 6.4 keV. **d-f** Corresponding images obtained with the FZP at its design energy of 6.2 keV.

References

[1] Chapman, H. N. & Bajt, S. High-resolution achromatic X-ray optical systems for broad-band imaging and for focusing attosecond pulses. *Proc. R. Soc. A* **477**, 20210334 (2021).

[2] Wang, Y., Yun, W. & Jacobsen, C. Achromatic Fresnel optics for wideband extreme-ultraviolet and X-ray imaging. *Nature* **424**, 50–53 (2003).

[3] Hecht, E. *Optics* (Addison-Wesley, 2001), 4 edn.

[4] Wakonig, K. *et al.* PtychoShelves, a versatile high-level framework for high-performance analysis of ptychographic data. *J. Appl. Crystallogr.* **53**, 574–586 (2020).
